# Maternal-Foetal/Infant Interactions—Gut Microbiota and Immune Health

**DOI:** 10.3390/biomedicines12030490

**Published:** 2024-02-22

**Authors:** Ada Maria Adamczak, Alicja Werblińska, Małgorzata Jamka, Jarosław Walkowiak

**Affiliations:** 1 Department of Pediatric Gastroenterology and Metabolic Diseases, Poznan University of Medical Sciences, 27/33 Szpitalna Street, 60-572 Poznań, Poland; a.adamczak@ump.edu.pl (A.M.A.); mjamka@ump.edu.pl (M.J.); 2 Greater Poland Centre for Pulmonology and Thoracic Surgery Named after Eugenia and Janusz Zeyland, 62 Szamarzewskiego Street, 60-569 Poznań, Poland; alicjawerblinska@gmail.com

**Keywords:** intestinal microbiota, immune system, pregnancy, childbirth, breastfeeding

## Abstract

In recent years, the number of scientific publications on the role of intestinal microbiota in shaping human health, as well as the occurrence of intestinal dysbiosis in various disease entities, has increased dynamically. However, there is a gap in comprehensively understanding the factors influencing a child’s gut microbiota. This review discusses the establishment of gut microbiota and the immunological mechanisms regulating children’s microbiota, emphasising the importance of prioritising the development of appropriate gut microbiota in a child from the planning stages of pregnancy. The databases PubMed, Web of Sciences, Cochrane, Scopus and Google Scholar were searched to identify relevant articles. A child’s gut microbiota composition is influenced by numerous factors, such as diet during pregnancy, antibiotic therapy, the mother’s vaginal microbiota, delivery method, and, later, feeding method and environmental factors. During pregnancy, the foetus naturally acquires bacterial strains from the mother through the placenta, thereby shaping the newborn’s immune system. Inappropriate maternal vaginal microbiota may increase the risk of preterm birth. Formula-fed infants typically exhibit a more diverse microbiota than their breastfed counterparts. These factors, among others, shape the maturation of the child’s immune system, impacting the production of IgA antibodies that are central to cellular humoral immune defence. Further research should focus on identifying specific microbiota–immune system interactions influencing a child’s immune health and developing personalised treatment strategies for immune-related disorders.

## 1. Introduction

The human gut microbiota is composed of trillions of microbes colonising the gastrointestinal tract, including bacteria, viruses, fungi, archaea, eukarya and parasites, of which bacteria are the dominant organisms [1,2]. The gut microbiota has co-evolved with humans and is an inseparable component of the human body [3]. Microbiota colonisation begins during foetal development and evolves alongside the host, maintaining its stability and diversity throughout life [4,5].

The intestinal microbiota consists of beneficial and pathogenic microorganisms which modulate many physiological processes and have various functions, including immune development [6], nutrient absorption [7], production of short-chain fatty acids (SCFAs) for energy metabolism [8] and vitamins (e.g., K and B12) [9], protection against pathogenic bacteria [10], and fat storage [11]; they may even impact human behaviour [12]. Thus, the gut microbiota is referred to as an essential organ [4,13].

The diversity and the quantitative and qualitative composition of the microbiota contributes to its stability, which in turn influences the appropriate functioning of all body systems. The balance of gut microbiota is upheld through feedback mechanisms, with a greater diversity regarded as being advantageous for overall health [14]. Reduced diversity has been linked to metabolic abnormalities, including inflammation, insulin resistance and dyslipidemia [1,15]. Inadequate intestinal microbiota in a child is associated with disturbed weight [16], necrotising enterocolitis (NEC) [17] or fungal infections, such as candidosis [18]. Importantly, the gut microbiome is essential in educating the immune system, as the early establishment of beneficial bacteria promotes immune tolerance, thereby reducing autoimmune diseases and food allergies [19]. The microbiome composition is dynamic and changes throughout life in response to various internal and external factors [4], such as the mother’s diet during pregnancy [20,21,22], the maternal health status [23], pre-pregnancy weight and subsequent weight gain [24], stress [25] and the use of antibiotics [26]. Significant postnatal factors include gestational age [27], mode of delivery [28], infant feeding method [29], and, later, medication intake [30,31,32], dietary habits [32,33], as well as the level of hygiene and environmental industrialization [34,35]. Studies unequivocally demonstrate that the microbiota composition may have implications for the nutritional, immunological, and microbiological aspects of a child’s health [36]. 

Recently, several papers have reviewed the influence of gut microbiota on the immune system. Méndez et al. [37] described the role of gut microbiota in shaping the immune system, primarily in the context of food allergies. Sanidad et al. [38] explored factors modulating a child’s gut microbiota and its impact on the immune system, but did not consider the potential consequences of dysbiosis in adulthood. Zhang et al. [39] discussed mutual correlations between gut microbiota and the immune system, focusing on the maturation process of both systems and considering influencing factors, as well as the potential consequences of their disturbance. In a review by Wiertsema et al. [8], detailed physiological interactions between gut microbiota and the immune system were highlighted, along with the influence of nutrition on modulating the microbiome and immune response, although this work was not focused on a specific social group.

This review discussed the impact of maternal microbiota on the course of pregnancy and the development of the foetal immune system, providing a comprehensive overview of the modulation of a child’s gut microbiota from conception to adulthood. Special attention was given to the role of maternal microbiota, which provides the child’s initial exposure to microorganisms and shapes its development throughout life. The focus is on analysing the mode of delivery on the composition of infants’ gut microbiota, assessing changes and their potential consequences for health in the first months of life. Additionally, attention was drawn to the feeding method and its impact on the composition of infants’ gut microbiota, aiming to identify potential significant effects on the child’s development during infancy. The final conclusion emphasises the role of gut microbiota in shaping the immune response, prompting considerations regarding the manipulation of this environment in the context of immunological diseases in children.

## 2. Methodology

A literature review was conducted from May to December 2023 of relevant articles assessing factors influencing gut microbiota, the interactions between maternal and foetal gut microbiota, and the role of gut microbiota in immune system development retrieved from PubMed, Web of Sciences, Cochrane, Scopus databases and Google Scholar. The search keywords were “intestinal microbiota”; “gut microbiota”; “immune system”; “immune system development”; “pregnancy”; “childbirth”; “delivery”; “child”; “breastfeeding”; and “diet”. Only articles published in the English language were considered. The focus was on studies involving human participants, and specific time frames were not defined to encompass all knowledge gathered to date, with the focus being on research from the last few years in particular. Books, documents and case reports were not included, and the search was restricted to meta-analyses, randomised controlled trials, observational studies, reviews and systematic reviews.

## 3. Influence of Maternal Microbiota on Pregnancy

Recent studies have confirmed that microbiota colonisation in the body begins during pregnancy, undermining the conventional notion of the foetus as a sterile organism [40]. Microbes are found in the placenta, amniotic fluid, umbilical cord blood and foetal membranes, underlining the importance of the mother’s normal microbiota during pregnancy as a key factor influencing pregnancy and the child’s health [5,41]. 

**Figure 1:**
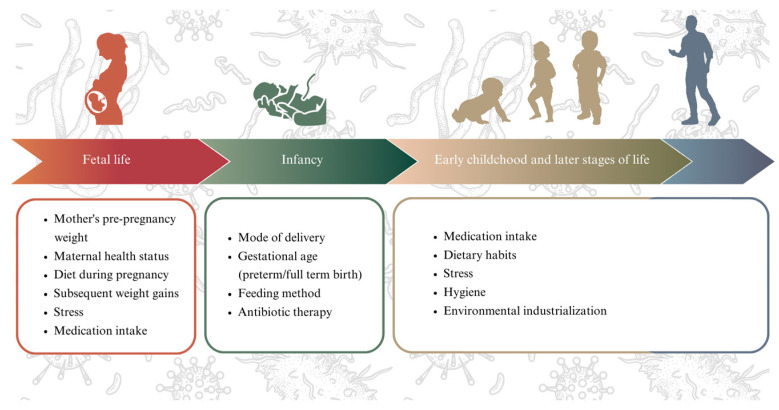
Overview of the factors influencing gut microbiome development.

Foetal immune system development is significantly influenced by the in-utero environment (Figure 1). 

The microbiota composition undergoes dynamic changes associated with significant hormonal, immunological, and metabolic transformations. These fluctuations are not limited to the gastrointestinal tract, including the oral cavity, but extend to the urogenital tract or skin. These changes are particularly pronounced in the third trimester when the microbiota composition is compared to a state of dysbiosis [42]. Typically, during this period, there is an increase in the *Lactobacillus* species such as *L. crispatus*, *L. jensenii*, *L. gasserii*, and *L. vaginalis* in the vaginal environment, and a decrease in anaerobic microorganisms such as *Atopobium*, *Prevotella*, *Sneathia*, *Gardenerella*, *Ruminococcaceae*, *Parvimonas* and *Mobilincus* [43]. Elevated levels of oestrogens contribute to the growth of the vaginal lining and increase the glycogen accumulation in the vaginal mucus [44]. *Lactobacillus* utilises glycogen to produce lactic acid and bacteriocins [41,45], protecting against vaginal dysbiosis and suppressing opportunistic infections. There is also a noticeable increase in the prevalence of Proteobacteria and Actinobacteria during the third trimester [21]. An abnormal vaginal microbiome in expectant mothers can influence the susceptibility to preterm birth [46]. 

A healthy vaginal microbiota also plays a crucial role in preventing vaginal bacterial infections, sexually transmitted diseases and urinary tract infections in the mother. Additionally, bacterial infections in the vagina reduce the abundance of the *Lactobacillus* species, significantly increasing the risk of sexually transmitted infections and potentially contributing to an increased likelihood of preterm birth [47]. Preterm infants face challenges in establishing their gut microbiota, as they are likely to experience a caesarean section (CS) delivery, antibiotic exposure, low birth weight and varying feeding patterns [48].

Nutritional elements within the maternal gut are metabolised by the intestinal microbiota to form molecular byproducts that are subsequently passed on to her offspring. The maternal diet can impact the composition and the transcriptional activity of the intestinal microbiota [49]. 

A typical feature of a full-term, healthy pregnancy involves metabolic changes to ensure that the foetus receives sufficient nutrients. These changes include increased insulin resistance to breakdown fats (lipolysis) and the conversion of glycerol molecules into glucose through gluconeogenesis, maintaining hyperglycaemia but increasing the circulating pro-inflammatory cytokines. During late pregnancy, the gut microbiota adjusts its genetic activity to make use of the increased glucose available in the bloodstream of the pregnant woman. Consequently, there is an increased production of enzymes and transporters required for processing glucose, while, at the same time, the activity related to other types of carbohydrates decreases [50].

Diseases related to glucose, lipid metabolism and obesity during pregnancy are significant indicators of adverse outcomes for both the mother and the newborn. There is a direct correlation between the mother’s gut microbiota and metabolic diseases, as the gut microbiota profile is associated with maternal obesity and hyperglycaemia [51].

The reduced richness of intestinal microbiota has previously been associated with elevated pro-inflammatory markers and insulin resistance [52]. Moreover, the gut microbiota in women with gestational diabetes mellitus (GDM) is similar to the microbiota observed in individuals with type 2 diabetes and related intermediary metabolic characteristics [53]. Previous pregnancies impact the maternal metabolic adaptation to subsequent pregnancies, and this extends to the adaptation of gut microbiota, which is directly linked to metabolism [54]. The human gut microbiome may maintain an ‘ecological memory’ from previous pregnancies, thereby reducing the limitations on the mother during subsequent pregnancies. 

Factors negatively affecting gut microbes, such as antibiotic therapy, low intake of natural probiotic strains and dietary errors, can, therefore, impact metabolic diseases, which can result in adverse pregnancy outcomes, such as preeclampsia, CS, preterm birth, or perinatal death [55]. Taylor et al. [56] concluded that probiotic supplementation leads to a significant reduction in insulin resistance in pregnant women diagnosed with GDM. Therefore, probiotic supplementation may be a potential therapy, supporting metabolism in cases of GDM. Additionally, women who regularly consume probiotic dairy products are expected to have a diminished likelihood of experiencing an unplanned preterm birth [57].

Dysregulation of the maternal and infant gut microbiota-associated immune system during the prenatal and postnatal periods is associated with profound and enduring neurodevelopmental consequences [58]. It is believed that the immune system and the gut microbiome have an interactive impact on the developing brain [59]. The diversity of microorganisms in the gastrointestinal microbiome (alpha diversity) in one-year-old infants was significantly correlated to amygdala–thalamus connectivity between the anterior cingulate cortex and insula, potentially resulting in behaviour modifications [60]. Jost et al. [61] examined the gut microbiota of seven healthy mothers during the perinatal period and found that, while the composition of the microbiota remained stable, there were fluctuations in the subdominant populations and increased metabolic activity, suggesting a potential impact of pregnancy on the gut microbiota.

Malnutrition during pregnancy impacts the immune function through a combination of direct and indirect mechanisms. A deficiency in essential nutrients reduces foetal white blood cells. Moreover, maternal malnutrition triggers stress responses in the mother and foetus, directly impeding placental function and foetal immune development. It also leads to maternal immunosuppression, reducing the availability of maternal antibodies for transmission to the foetus and making the mother more susceptible to infections [62]. Intrauterine growth retardation is associated with an elevated susceptibility to postnatal infectious diseases [63].

## 4. Impact of Maternal Microbiota on Foetal Immune System Development

Infants have underdeveloped immune systems at birth and therefore rely on the mother for immediate protection [64]. Cytokines are produced only at low levels, and many cells, such as phagocytes and dendritic cells, are not yet adequate in number and function, with a limited lymphocyte population. The primary driving force behind the growth of the lymphoid population is the encounter with the microbial community inhabiting the intestinal tract [65].

Previously, it was widely accepted that birth was the initial opportunity for the mother’s microorganisms to colonise the infant’s gut and influence the immune system [66]. However, recent findings have revealed that this interaction between the maternal microbiota and the infant’s immune system begins considerably earlier during pregnancy due to the presence of gut microorganisms in the placenta, amniotic fluid, and foetal membranes [67,68,69]. Research has demonstrated that the mother’s nutritional status plays a pivotal role in the developmental programming and alteration of the risk of non-communicable diseases in their offspring through epigenetic modifications [70].

There is a growing acknowledgement that the gut microbiome not only governs the local mucosal immune system but also impacts systemic immune responses involving both innate and adaptive cell-mediated pathways through various mechanisms [71]. Between the first and seventh days after birth, there is a swift and organised proliferation of the intestinal microbiome to establish a relatively mature microbial ecosystem, primarily dominated by Proteobacteria, Firmicutes, Bacteroidetes, and Actinobacteria by the seventh day post-birth [72]. Simultaneously, there is an exponential increase in neutrophils, the immune cells within the gut. This surge is accompanied by the maturation of monocytes and macrophages, which reach their full development around the seventh day after birth [73].

Various maternal variables, such as antibiotic use, diet, obesity and diabetes, have been linked to alterations in the establishment and evolution of their offspring’s gut microbiome, which affects its immunity [74]. Infants born to mothers with *Lactobacillus* colonisation in the vaginal area exhibited decreased levels of interleukin (IL) 12 in the umbilical cord blood after delivery. IL-12 enhances the cytotoxic response of natural killer (NK) cells and macrophages. Furthermore, *Lactobacilli* inhabiting the maternal vaginal microbiota exert a notable influence on the modulation of neonatal T cell development. CD4+ T helper cells (CD4+) and CD8+ T lymphocytes (CD8+) are present in the foetus towards the end of the first trimester. These lymphocytes produce IL-2 and a tumour necrosis factor (TNF)-α, as well as support intestinal development. These findings suggest that the presence of *Lactobacillus* in the maternal vagina has an impact on the development of the foetal immune system [75,76].

The authors of the Barwon Infant Study [77] discussed the impact of the maternal diet and the presence of *Prevotella* during pregnancy on the likelihood of the offspring developing food allergies. They discovered a substantial, dose-dependent association between the presence of maternal *Prevotella* in stools and a decreased risk of food allergies in 12-month-old infants. The gut microbiome serves as a repository for antibiotic resistance genes (ARGs), and the infant microbiome is more vulnerable to external influences than the more stable adult microbiome. In neonates, the intestinal environment offers lower colonisation resistance, potentially facilitating the establishment of antibiotic-resistant populations. Factors correlated with an increased ARG prevalence include antibiotic use during childbirth and in the neonatal period [78]. Conversely, breastfeeding and the administration of probiotics to neonates are linked with a reduced presence of ARGs [79]. The meta-analysis by Grech et al. [80] demonstrated that maternal exposure to antibiotics during childbirth was associated with a modest reduction in alpha diversity in their newborns as compared to that in the control group, who were not exposed to antibiotics.

## 5. Impact of Delivery Mode on the Microbiota Composition in Infants

One of the key factors influencing the diversity and colonisation of the gut microbiota in infants is the mode of delivery [28,81]. Shao et al. [82] showed that the type of delivery is the strongest perinatal factor influencing the child’s microbiota among other clinical covariates, such as perinatal antibiotic therapy, length of hospital stay or breastfeeding. The colonisation dynamics of the gut microbiota in infants born via CS are distinct from those born vaginally, particularly during the first six months of a child’s life [28]. The modified gut microbiota of infants delivered via CS may elevate susceptibility to conditions such as food allergies [83] or coeliac disease [84]. Recent meta-analyses documented a correlation between CS affecting gut microbiota and higher risks of asthma [85], diabetes [86], excess weight and obesity [87]. 

The primary cause of this phenomenon is likely the lack of exposure of the child to the mother’s gut and vaginal microbiota during childbirth. This exposure plays a crucial role in shaping the child’s immune system development, and its absence can disrupt optimal immune system development, leading to an increased risk of the aforementioned diseases [28]. Infants delivered vaginally acquire bacterial communities that closely resemble mothers’ vaginal microbiota, primarily consisting of *Lactobacillus*, *Prevotella* or *Sneathia* spp., whereas infants delivered by CS harbour bacterial communities resembling those present on the skin surface, predominately *Staphylococcus*, *Corynebacterium* and *Propionibacterium* spp. [88]. Both CS delivery and the administration of antibiotics during labour impact the infant’s gut microbiota, as they reduce the *Bacteroides* levels, irrespective of the infant’s diet during the initial three months of life [89]. Additionally, exclusive breastfeeding has a beneficial effect on the recovery of Actinobacteria and *Bifidobacteria* during the first three months after birth [90]. Over time, the influence of the delivery mode on a child’s microbiota development becomes less prominent, while the most important modifications to the gut microbial communities are primarily shaped by feeding practices, stress, previous infections, medication intake and environmental aspects [91].

During the initial days of a child’s life, the predominant bacterial species in the oral cavity and gastrointestinal tract include *Gardnerella vaginalis*, *Propionibacterium acnes*, *Prevotella bivia*, *Atopobium vaginae* and *Prevotella melanino genica*, which tolerate higher pH levels than the acidophilic *Lactobacillus* species commonly found in the maternal gut. In contrast, the pH of the gut of a newborn is closer to a neutral value. From the third day of a child’s life, specific bacterial strains, including *Rothia mucilaginosa*, *Streptococcus parasanguinis* and *Streptococcus salivarius* become increasingly prevalent. The microorganisms that colonise the infant, derived from the mother’s intestinal tract, exhibit remarkable resilience and optimal adaptability. Nonetheless, bacteria sourced from other maternal origins, including the vagina and skin, exert a transient yet influential impact on the establishment of the child’s microbiota [92].

The Finnish Birth Cohort study analysed asthma prevalence in seven-year-old children and discovered that those born by vaginal delivery exhibited reduced asthma rates than the children delivered via CS [93]. A Dutch cohort investigation, the KOALA study, revealed that the mode of childbirth had a direct impact on the risk of developing asthma and atopic manifestations [94]. In the Turku Birth Cohort, a higher frequency of positive allergy test results was observed in the CS delivery group than in the group of infants delivered vaginally [93]. In a study involving more than 1000 infants, infants delivered by CS had lower levels of *Bifidobacterium* and *Bacteroides* spp. in their stool samples while exhibiting higher levels of pathogenic microorganisms, including *Clostridium difficile*, than infants born vaginally. This biological relationship may serve as a precursor to dysbiosis [95]. Similar results were also observed in a study of 98 infants (including 15 born via CS), which revealed that infants born via CS exhibited a higher proportion of *Clostridium* spp. at 4 months and 1 year of age [13]. Princisval et al. [96] documented that infants delivered by CS exhibit a lower degree of colonisation by the *Bifidobacterium* (*B. longum*, *B. catenulatum*) and *Bacteroides* species (particularly *B. fragilis*, *B. vulgatus*, and *B. uniformis*), as well as *Lactobacillus* and *E. coli*, but were more heavily colonised by *Clostridium* (*C. perfringens*). Furthermore, infants delivered by CS are more frequently colonised by pathogenic bacteria [82]. Of notable significance is the *Bacteroides* genus, which tends to be present in infants born through vaginal delivery and shared with their mothers. Although the *Bacteroides* strains are more prevalent in mothers of infants delivered by CS after 6 months, the sustained decrease in these shared strains in CS-born infants suggests the need for further research into potential deficits in the microbiota establishment and long-term impacts at the level of individual bacterial taxa [97].

In a study involving a cohort of 25 healthy women who underwent uncomplicated vaginal deliveries, resulting in healthy full-term infants, it was observed that the most significant contributors of acquired microbial strains in neonates were derived from the maternal gut microbiota, followed by the vaginal microbiota. The microbes from the breasts and the infant’s oral cavity appeared to have a comparatively minor role in the transmission process. Furthermore, a gradual decline in the number of shared strains between the mother and the child was observed over the postnatal period [98]. In children born via CS, a ‘baby seeding’ method is sometimes used to compensate for the lack of natural microbiota transfer from the mother, and it involves transferring maternal vaginal microbiota to the newborn shortly after birth. In a double-blind, randomised, placebo-controlled trial conducted on 20 newborns, vaginal seeding induced changes in the skin and stool microbiota, leading to a shift in bacterial diversity resembling that found in vaginally born and breastfed infants. Vaginal seeding reduced the presence of several potential pathogens, such as *Enterobacter*, in the transitional stool and *Clostridium* spp. in the stool on the 30th day. Additionally, an increase in *Lactobacillus* was observed on the newborns’ skin [99].

Another study demonstrated that post-birth transfer of maternal vaginal microbiota does not constitute an effective method for preventing allergies in children during the first two years of life. For infants subjected to vaginal seeding, the diversity of the *Lactobacillus* and *Bacteroides* species was slightly, but insignificantly, elevated in the gut microbiota after birth and at 6 months of age. The results also revealed that the risk of being overweight and of obesity was lower at 6 months of age, but a similar relationship was not observed for other periods [100].

## 6. Impact of Feeding Method on the Infant Gut Microbiota Composition

Beyond the mode of childbirth, a significant determinant in shaping the infant’s gut microbiota is the feeding practice, as well as the introduction of solid foods into the child’s diet. The feeding method influences the abundance and diversity of infant microbiota, which modulates the immune system response [29]. Breast milk has been acknowledged as the optimal source of nourishment for infants, providing a wide array of nutrients and bioactive components that contribute to their overall growth and development [101]. Human breast milk harbours a unique and diverse microbial community [21] with the gut microbiome of breastfed infants primarily populated by *E. coli* and *Bifidobacterium* spp. (*B. longum*, *B. breve*, and *B. bifidum*). Up to the age of 16 months, the child’s microbiome contains a significant amount of bifidobacteria, indicating that their gut microbiota is similar to that of an infant, whereas adults generally have a low abundance of *bifidobacteria* [98]. There are three hypothetical routes for breast milk colonisation by microorganisms. The first assumes intracorporeal translocation from the maternal gut microbiota to the mammary gland through dendritic cells [102,103], the second refers to the imbibition of microorganisms from the mother’s skin during breastfeeding [104], while the third relates to the form of delivery, as the child born naturally has microorganisms on its skin from the mother’s birth canal, which colonise the nipple [102,104,105]. Jost et al. [106] revealed that viable strains of obligate anaerobic gut-associated bacteria, such as *Bifidobacterium*, *Bacteroides*, *Parabacteroides* and *Clostridia* (*Blautia*, *Clostridium*, *Collinsella* and *Veillonella*) are shared between the mother and newborn through breastfeeding, indicating a form of mother–infant communication. Maternal gut bacteria reach breast milk and influence the colonisation of the infant’s gut and immune system maturation [107]. Breast milk not only provides infants with beneficial microbiota for gut colonisation but also contains immunological components that protect against infections and allergies, such as lactoferrin, secretory immunoglobulin A (sIgA), transforming growth factor-β (TGF β) and α-lactalbumin [108]. Additionally, the presence of human milk oligosaccharides (HMOs) and prebiotic complex sugars promotes the growth and activity of beneficial bacteria, such as *Bifidobacterium*, *Lactobacillus* and *Bacteroides*, that decompose these oligosaccharides into smaller sugars for a readily available energy source [109]. This provides them with a competitive advantage over other bacterial species [29,110]. The presence of nutritional components, immunological factors, hormones, HMOs, and microbiota in breast milk makes it the most suitable food for a developing organism [111]. Significantly, notable disparities have been documented in the intestinal microbiota composition between breastfed neonates and those fed with commercial infant formula. The research underscores that infants receiving breast milk exhibit a diminished diversity in their microbiota than those receiving standard formula or formula incorporating isolated soy protein. Moreover, infants fed with soy protein have decreased levels of *Bifidobacterium* and elevated *Ruminococcaceae* compared to their breastfed counterparts [112]. It is believed that increased bacterial diversity might be beneficial for adults, while decreased diversity in breastfed infants appears to be advantageous for the immature gastrointestinal and immune systems [112,113]. In a study conducted on a group of 78 full-term infants who were breastfed or formula-fed (standard formula or *L. paracasei*-enriched semi-fermented milk), it was observed that newborns born naturally and fed a semi-fermented formula had increased *Ruminoccocus* and *Erysipelotrichaceae* bacteria and less *Bacteroides* and *Parabacteroides* in stool samples. Infants fed standard formula, irrespective of the type of delivery, had a higher amount of *Clostridium innocum*. In addition, children born naturally and fed a partially fermented formula had higher concentrations of sIgA in the blood [114]. A study conducted on a group of 33 preterm infants, divided into six groups (1. exclusively breastfed, 2. fed with human donor milk, 3. fed with formula milk, 4. breastfed + formula milk, 5. breastfed + human donor milk, and 6. human donor milk + formula milk), revealed that preterm infants fed with maternal milk (at least 70%) had more *Clostridiales*, *Lactobacillales*, and *Bacillales*, whereas *Enterobacteriales* was predominant in infants fed with formula milk or milk from a human milk bank [115]. Sjödin et al. [116] suggested that enriching infant formulas with specific synbiotics (fructo- and galactooligosaccharides from *Lactobacillus paracasei* ssp. *paracasei F19*) is more beneficial for infants than using prebiotics alone (fructo- and galactooligosaccharides). Administering synbiotic-enriched formulas to infants resulted in the prevention of a significant *Klebsiella* colony increase, but there were increased *Bifidobacterium* breve colonies and metabolite d-3-phenyllactic acid, which exhibited antibacterial properties. This metabolite also regulates immune signaling in the gut–lung and gut–skin axes [116]. In another study, enriching milk with bovine milk-derived oligosaccharides supported the growth and proliferation of bacteria from the *Bifidobacterium* genus, reducing the presence of foecal pathogens and enhancing the intestinal immune response, thereby exerting a beneficial influence on the developing microbiome [117]. Xu et al. [118] confirmed that the most beneficial source of nutrition and support for the gut microbiome in preterm infants is the mother’s breast milk. Variations in the fatty acid composition resulting from different feeding methods can influence the types of microbes present. Ongoing research is assessing the potential of adding prebiotics and probiotics to promote a healthy gut microbiome. Disparities in the distribution of immune cells in newborns based on their diet can be discerned in up to six months of life, with natural killer cells exerting the most significant influence [118]. In infants fed with formula, the maturation of their immune system predominantly leans towards cells exhibiting acquired immunity, while the progression of innate immunity is comparatively delayed in formula-fed infants than in breastfed infants [119].Various types of early life milk feeding exert distinct effects on the child’s gut microbiota. Table 1 provides a comparison of selected studies examining the effects on the composition of gut microbiota of various milk formulas.

## 7. Role of Infant Gut Microbiota in the Immune Response

The gut microbiota has unique immunomodulatory capabilities, conditioning the appropriate development of immune response, which translates into a reduced risk of autoimmune diseases [19]. The first three months of an infant’s life are particularly crucial, as qualitative and quantitative changes in the gut microbiota influence the immune system development, with bacterial diversity being more significant than specific bacterial taxa [126]. The gut microbiota composition has profound implications on the circulating cytokines (such as IL-12p40, interferon γ, TGFβ, IL-β, IL-6, TNF-α and monocyte chemoattractant protein-1) and lymphocyte development, as some bacterial species stimulate the differentiation of specific T-cell subtypes [127]. *Bifidobacteriaceae* may play a key role in immunomodulation. Studies report that infants whose intestines are not colonised by this bacterial species or do not acquire these microorganisms in the first months of life are more susceptible to systemic inflammation, including intestinal inflammation. A decreased abundance of *Bacteriaceae* in infants was associated with an increased number of neutrophils, basophils, and CD8+ T lymphocytes [128]. Commensal gut microbes play a crucial role in regulating intestinal mucosal immune system maturation, promoting homeostasis, while pathogenic microorganisms contribute to the disruption of this delicate balance. Dysbiotic disturbances in the gut microbiota can trigger the onset of atopic diseases [129], food sensitivities [130], necrotising enterocolitis [129], or inflammatory bowel diseases [10,110]. Recent studies have also revealed the direct mechanisms dictating the mutualism between the microbiome and the immune system. For example, B cells play a crucial role as mediators of gut homeostasis [10] and impact the IgA secretion in the human intestines, which form the basis of mucosal humoral immune protection [127,131]. The IgA action in the gut contributes to the maintenance of a diverse and balanced microbiome, facilitating the expansion of Foxp3+ regulatory T (Treg) cells that secrete anti-inflammatory interleukins. IgA also has a protective function as it coats colitogenic bacteria preventing inflammation [132]. Another example of microbiome regulation involves CD8+ (cytotoxic) T cells which eliminate intracellular pathogens and cancer cells [10]. In turn, sIgA, plays a pivotal role in orchestrating the training and enhancement of the immune system, as it specifically targets a broad array of intestinal bacteria, influencing the promotion or restriction of bacterial species, a crucial mechanism in facilitating the diversity within the gut microbiome [133].

The human immune system has innate and adaptive immune responses, constantly interacting with the microbiota. The innate immune response maintains homeostasis by eliminating pathogenic bacteria involving factors such as IgA, Toll-like receptor 5, autophagy and inflammasome cytokines [134,135,136]. The adaptive immune response is also important for maintaining an appropriate microbiota and immune balance. Education of immune response is achieved through the differentiation of T and B lymphocytes, as well as the stabilisation of the immune response to specific commensal microbes [137]. The intestinal microorganisms also determine the differentiation of Th1, Th2, and Th17 lymphocytes [138]. Furthermore, the gut microbiota is closely related to the immune response, as gut microbes can transport macromolecules and antigens across the gut epithelium [139]. Differentiated by the action of flagellin (a protein that builds the bacterial flagellum), B cells produce IgA, which neutralises pathogens, thus reducing the risk of infection [140]. The intestinal microbiome also contributes to immunity through the lymphoid tissue directly related to the intestines, including Peyer’s patches, plasma cells and lymphocytes [141]. Other studies have shown that gut bacteria interact with the mucosa, which secretes antibodies that are then captured by the dendritic cells in Peyer’s patches [140,142]. 

There is recent evidence that the gut microbiota composition may also influence the development of allergies [143]. Infants born via CS have a higher risk of developing allergies than those born vaginally [144]. Common characteristics have been observed in the microbiome of children diagnosed with allergies, whether in terms of food or respiratory aspects. There are increased *Firmicutes* bacteria and decreased *Bacteroidetes* in the gut; in particular, *Ruminococcus gnavus* bacteria in allergic individuals more readily adhere to the intestinal epithelium, which may contribute to developing a pathogenic mechanism [143]. Furthermore, the gut microbiome of allergic children exhibits an increased expression of genes involved in lipopolysaccharide biosynthesis, which stimulates the production of pro-inflammatory cytokines [145]. A comprehensive review involving six studies that examined the influence of probiotic therapy on a wider spectrum of allergic diseases showed that probiotics may potentially decrease the likelihood of atopic dermatitis or eczema in infants when administered to mothers during pregnancy and the initial 36 months after childbirth. Notably, treatment involving a blend of probiotic strains such as lactobacilli and bifidobacteria can be efficacious, particularly in cases where the child has a heightened genetic predisposition to allergic conditions [146]. A randomised controlled trial of 101 infants born via CS in China assessed the effectiveness of the *Lactobacillus paracasei N1115* strain in improving gut development, showing that this strain increased the Lactobacillus content and the sIgA levels in the stools while reducing salivary cortisol levels in both infants and children. The latter was more pronounced in infants aged 6–12 months [147]. In a placebo-controlled, double-blind, randomised trial involving 110 healthy full-term infants, it was demonstrated that supplementation with *Bifidobacterium longum subsp. infantis M-63* decreased stool pH and increased foetal acetic acid and IgA levels at 1 month. The supplementation primarily influenced the genus *Bifidobacterium*. These findings indicated that administering *B. infantis M-63* at a dose of 1 billion per day to healthy full-term infants has a beneficial effect on maintaining infant health by promoting the development of the intestinal microbiota [148].

SCFAs, including propionic, butyric, and acetic acid, also play a significant role in immune system regulation and are the most abundant microbial metabolites in the colon. SCFAs are utilised by colonocytes as a primary source of energy and influence the expression of genes essential for maintaining the epithelial barrier function and immune defence. They also regulate the function of innate immune cells, such as macrophages and neutrophils [149]. Additionally, they impact antigen-specific acquired immunity mediated by T and B lymphocytes [131]. Butyric acid and other SCFAs exert direct anti-inflammatory effects on the intestine [150].

The control of immune response against self-antigens is primarily orchestrated by Treg cells, a specialised subset of CD4+ T cells known for their immunosuppressive properties. These cells play a significant role in directing adaptive immune reactions, generating effector cytokines that fulfill various roles in the immune system. SCFAs stimulate the generation and proliferation of Tregs, which are crucial to preventing inflammation during foetal development and in protecting against the rejection of semi-allogeneic maternal cells or tissues. They achieve this by supressing pro-inflammatory T cells activity and promoting tolerance toward maternal antigens [151].

SCFAs exert their anti-inflammatory and immune-modulating effects through two primary pathways. First, signalling through G-protein coupled receptors on target cells. Second, they inhibit histone deacetylases, affecting gene expression regulation and demonstrating anti-inflammatory effects in the colonic mucosa [151,152]. In patients with inflammatory bowel diseases (IBD), a reduction in bacterial biodiversity, notably in Bacteroides and *Firmicutes*, may lead to decreased butyric acid production [153]. However, probiotic strains of SCFA-producing *Bifidobacterium* and *Lactobacillus* have been shown to alleviate IBD in human models by enhancing the intestinal epithelial barrier and modulating the immune response [152].

## 8. Conclusions

In conclusion, the gut microbiota directly impacts the child’s health development, beginning in the prenatal period. Changes in the gut microbiota are influenced by the mother’s diet during pregnancy, potential perinatal antibiotic therapy, mode of delivery, feeding method, and the intake of medications and supplements, as well as the diet, hygiene and the level of environmental industrialisation later in life. An optimal composition of the maternal vaginal microbiota, enriched with the *Lactobacillus* species, confers protection against infections that could precipitate preterm labour. Moreover, disturbances in the maternal gut microbiota, causing factors like antibiotic therapy or dietary errors, can lead to metabolic diseases. These imbalances are potentially leading to adverse pregnancy outcomes, including preeclampsia or preterm birth. Throughout gestation, the foetus acquires bacterial strains from the mother via the placenta, which influence the maturation of the neonate’s immune system. Additionally, the mode of delivery significantly impacts the infant’s microbial colonisation with vaginal born infants, having reduced levels of pathogenic bacteria, including *Clostridium difficile*, which can act as precursors to dysbiosis and increased strains that prevent infections, such as *Lactobacillus*, *Prevotella*, or *Sneathia* spp. Moreover, the feeding modality shapes the trajectory of microbiota development and immune system maturation, with formula-fed infants exhibiting a more diverse microbiota profile, characterised by a higher proportion of anaerobic bacteria, in contrast to their breastfed counterparts, likely due to the dominance of *Bifidobacterium.* The gut microbiota substantially modulates immunological responsiveness through its impact on the IgA antibody production, which is the cornerstone of cellular humoral immune defence, impacting the risk of autoimmune diseases. The first three months of a child’s life are particularly crucial, as changes in bacterial diversity during this time shape the maturation of the immune system more significantly than the presence of specific bacterial species. Therefore, it is essential to prioritise the appropriate, healthy development of the gut microbiota, commencing from the earliest stages, even during preconception planning.

## 9. Future Directions

Enhancing our understanding of microbiota–immune system interactions is crucial for protecting the development of a child’s immune system. This knowledge can also assist in creating targeted interventions and deepen our comprehension of how individuals respond differently to treatments. Ultimately, this could pave the way for precision approaches to treating a variety of immune-related disorders in the future.

## Figures and Tables

**Table 1:** A comparison of selected studies examining the effects on the composition of gut microbiota of various milk formulas.

Authors	Year	Design	Group		*n*	Age (Inclusion Criteria)	Intervention Duration	Outcome
Rinne et al. [120]	2005	CoS	IG	Breastfeeding + probiotic (*B. lactis BB 12*)	8	6 months	6 months	Total number of *bifidobacteria* was lower among the formula-fed group than in other groups (*p* = 0.044). Total amounts of the other bacteria were comparable between the groups. The specific *Bifidobacterium* microbiota composition of the breast-fed infants was achieved in infants receiving prebiotic-supplemented formula.
				Formula feeding + prebiotic (a mixture of fructo (10%) and galacto-oligosaccharides (90%))	8			
			CG	Breastfeeding	8			
				Partially hydrolysed formula feeding	8			
Hascoet et al. [121]	2011	RCT-DB	IG	Study formula (low protein and phosphate content, high lactose and contained predominantly whey protein compared with the control formula)	39	≤7 days	>4 months	*Bifidobacteria* counts were significantly higher in infants receiving the study formula alone (median 10.0, interquartile range [IQR] 0.8, *p* < 0.0001) or with BL999 (median 9.8, IQR 1.4, *p* < 0.01) compared to the control group (median 9.2, IQR 3.5). These counts were similar to those in breast-fed infants (median 10.1, IQR 0.4, *p* > 0.05). The difference between the study groups was 0.16 log CFU/g, with a 90% confidence interval (CI) of 0–0.4, falling within the predefined equivalence margin. The microbiota profile, expressed as a percentage of total bacterial counts, indicated about 50% *Bifidobacteria*, 8% *Enterobacteria* and less than 10% *Clostridia* in both the study formula-fed and breast-fed infants, versus 22%, 13% and 19% in the control group, respectively. There were no significant differences in growth measurements, digestive tolerance or adverse events between the groups.
				Study formula + *B. longum BL999* (the same as the study formula but supplemented with 2 × 10^7^ colony-forming units (CFU)/g of B longum, strain BL999	40			
			CG	Control formula	38			
			RG	Breastfeeding	73			
Tannock et al. [122]	2013	RCT	IG	Goat milk formula	30	<2 weeks	2 months	Beta-diversity analysis of total microbiota sequences and *Lachnospiraceae* sequences revealed that they were more similar in breast milk/goat milk comparisons than in breast milk/cow milk comparisons. The *Lachnospiraceae* were predominantly restricted to a single species, *Ruminococcus gnavus*, in both breast milk-fed and goat milk-fed babies, as opposed to a more diverse collection observed in cow milk-fed babies. *Bifidobacteriaceae* were abundant in the microbiotas of infants across all three groups. *Bifidobacterium longum*, *Bifidobacterium breve*, and *Bifidobacterium bifidum* were the most commonly detected bifidobacterial species. A semi-quantitative PCR method was developed to differentiate between *B. longum* subsp. *longum* and *B. longum* subsp. *infantis*, which was subsequently employed to test stool samples. *B. longum* subsp. *infantis* was infrequently present in stools, even in those of breast milk-fed babies. The presence of *B. bifidum* in the stools of breast milk-fed infants, at abundances greater than 10% of the total microbiota, correlated with the highest total abundances of *Bifidobacteriaceae*. Conversely, when *Bifidobacteriaceae* abundance was low, *Lachnospiraceae* abundances were higher.
			CG	Cow milk formula	30			
			RG	Breast milk	30			
Zanella et al. [123]	2019	CoS	Exclusive breast milk		7	Newborns (gestational age ≤ 32 weeks)	28 days	There were significant differences in the microbial community among treatments. Approximately 37% of the variation in distance between microbial communities was explained by the use of exclusively mother’s milk only compared to other diets. The diet composed of exclusively mother’s milk allowed for greater microbial richness (average of 85 OTUs) while diets based on preferably formula, exclusive formula, preferably maternal milk, and mixed formula and maternal milk presented an average of 9, 29, 23, and 25 OTUs, respectively. The mean proportion of the genus *Escherichia* and *Clostridium* was always greater in those containing formula than in those with maternal milk only.
			Exclusive formula (Pre Nan)		8			
			Predominance of breast milk (>70% own mother’s milk)		16			
			Predominance of formula (>70% preterm formula)		16			
			Mixed (50% own mother’s milk and preterm formula)		16			
Kok et al. [124]	2020	RCT-DB	IG	Free amino acid-based formula	25	≤7 days	2 months	The relative abundance of *Bifidobacterium* increased over time and was significantly enriched at the end of the intervention in the breast-fed group. In contrast, a significant increase in members of the *Firmicutes* was detected in the study formula groups at the end of the intervention, along with an increase in butyrate-producing species in the cow milk-based, hydrolysed formula group. Stool pH was significantly higher in the free amino acid-based formula group both midway and at the end of the intervention. There was a significant increase in butyrate from baseline to the end of the intervention in the cow milk-based, hydrolysed formula group and butyrate levels were significantly higher in the study formula groups compared to the breast-fed group at the end of the intervention.
				Cow milk-based formula (extensively hydrolysed)	28			
			CG	Breastfeeding	25			
Brink et al. [112]	2020	RCT	3/6/9/12 months	Breastfeeding	16/20/12/14	1–2 months	~12 months	At 3, 6 and 9 mo of age, breast-fed infants had the lowest α-diversity, soy-based formula-fed infants had the highest diversity, and dairy milk-based formula-fed was intermediate. *Bifidobacterium* was 2.6- to 5-fold lower in soy-based fed relative to breast-fed infants through 1 y of life. In breast-fed infants higher levels of butyric acid, d-sphingosine, kynurenic acid, indole-3-lactic acid, indole-3-acetic acid, and betaine were observed than in dairy milk-based formula fed and soy-based formula-fed infants.
				Dairy-based milk formula	1219/11/14			
				Soy-based milk formula	14/15/12/15			
Ma et al. [125]	2020	CoS	IG	Formula A-feeding (containing α lactalbumin and β casein)	30	40 days	4 months	Among the different groups, α-diversity was lower in the breast-fed group compared to the formula-fed groups at 40 days of age. However, it increased significantly at 6 months of age. *Bifidobacterium* was the most predominant genus, and *Enterobacteriaceae* was the second most predominant in all groups.
				Formula B-feeding ((containing α lactalbumin, β casein, as well as 1, 3-Olein2-Palmitin)	31			
			CG	Breastfeeding	30			

RCT: randomized controlled trial; DB: double-blind; CoS: comperative study, *n*: sample size, IG: intervention group, CG: control group, RG: reference group.

## Data Availability

Not applicable.

## Abbreviations

ARGs—antibiotic resistance genes; CS—caesarian section; CoS—comparative study; CT—controlled trial; DB—double-blind; GDM—gestational diabetes mellitus; HMOs—human milk oligosaccharides; IBD—inflammatory bowel diseases; IG—intervention group; IL—interleukin; NEC—necrotizing enterocolitis; NK—natural killer; n—sample size; PS—prospective study; RG—reference group; RCT—randomised controlled trial; SCFAs—short-chain fatty acids; sIgA—secretory immunoglobulin A; TGF β—transforming growth factor-β; TNF-α—tumour necrosis factor-α; Treg—regulatory T cells.
